# Crystal structure of bergapten: a photomutagenic and photobiologically active furan­ocoumarin

**DOI:** 10.1107/S2056989016011221

**Published:** 2016-07-22

**Authors:** A. K. Bauri, Sabine Foro, Quynh Nguyen Nhu Do

**Affiliations:** a Bio-Organic Division, Bhabha Atomic Research Centre, Trombay, Mumbai 400 085, India; b Institute of Materials Science, Darmstadt University of Technology, Alarich-Weiss-Strasse 2, D-64287 Darmstadt, Germany; c Accident & Emergency Department, Franco, Vietnamese Hospital, 7-Nguyen, Luong Bang Street, HoChiMinh City, Vietnam

**Keywords:** crystal structure, bergapten, *T. sticto­carpum*, psoralen, furan­ocoumarin, photobiological activity, C—H⋯O hydrogen bonds, π–π inter­actions

## Abstract

The title compound, C_12_H_8_O_4_, is a furan­ocoumarin [systematic name: 4-meth­oxy-7*H*-furo[3,2-*g*]chromen-7-one], which was isolated from the Indian herb *T. stictocarpum*. The mol­ecule is almost planar with an r.m.s. deviation of 0.024 Å for the hetero atoms of the fused-ring system. In the crystal, mol­ecules are linked by C—H⋯O hydrogen bonds, forming a three-dimensional framework. There are offset π–π inter­actions present involving the coumarin moieties stacking along the *a*-axis direction [shortest inter-centroid distance = 3.717 (3) Å].

## Abstract 2

The title mol­ecule, bergapten, a psoralen/furan­ocoumarin derivative, possesses photocarcinogenic and photomutagenic activity. In the crystal, mol­ecules are linked *via* C—H⋯O hydrogen bonds, forming a three-dimensional framework.

## Chemical context   

The title mol­ecule, bergapten, is a linear furan­ocoumarin having a meth­oxy group in the benzene ring at position C5. This class of furano coumarins have absorption bands in the near UV region due to the presence of conjugated double bonds, and exhibit photomutagenic (Appendino, *et al.*, 2004 ▸) and photocarcinogenic properties, binding with purine bases of DNA in living cells to yield photoadducts (Filomena *et al.*, 2009 ▸). Based on this property, they are employed to treat numerous inflammatory skin diseases, such as atopic dermatitis, and pigment disorders like vitiligo and psoriasis by UV photodynamic therapy. In addition, due to their strong ability to absorb UV radiation, this class of mol­ecules are utilized as photoprotective agents, to prevent the absorption of harmful UV radiation by the skin. A variety of sun-screen lotions are widely used in dermatological applications in the cosmetic and pharmaceutical industries (Chen *et al.*, 2007 ▸, 2009 ▸). In addition, the *in vitro* anti­proliferation activity and *in vivo* photoxicity of the title mol­ecule has been reported against epithelial cancer cell lines, including HL60, A431 (Conconi *et al.*, 1998 ▸). Bergapten (5-meth­oxy psoralen/methoxsalen) has been used successfully in combination with UV photodynamic therapy to mange psoriasis and vitiligo; it inhibits proliferation in human hepatocellular carcinoma cell line (March *et al.*, 1993 ▸). Experimental results revealed that its phototoxicity and photomutagenicity is exerted *via* a Diels–Alder reaction binding the double bond of a purine base of DNA in a living cell with the double bonds of bergapten to yield mono- and di-adducts (Conforti *et al.*, 2009 ▸).
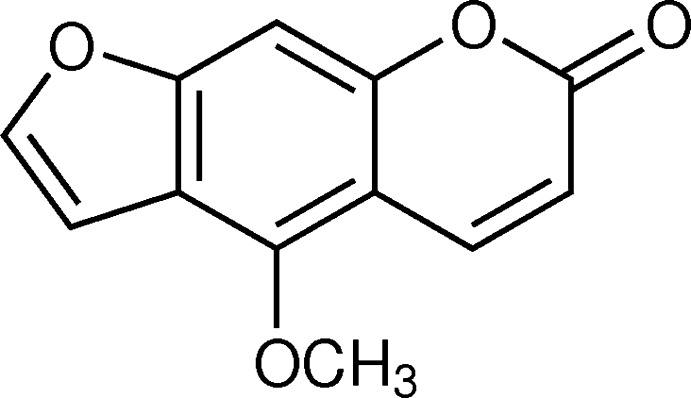


While this is the first report of the crystal structure of the title compound, its chemical structure was determined by spectrometric and spectroscopic analysis many years ago (Howell & Robertson, 1937 ▸; Ray *et al.*, 1937 ▸; Lin *et al.*, 1979 ▸; Confalone & Confalone, 1983 ▸).

## Structural commentary   

**Figure 1:**
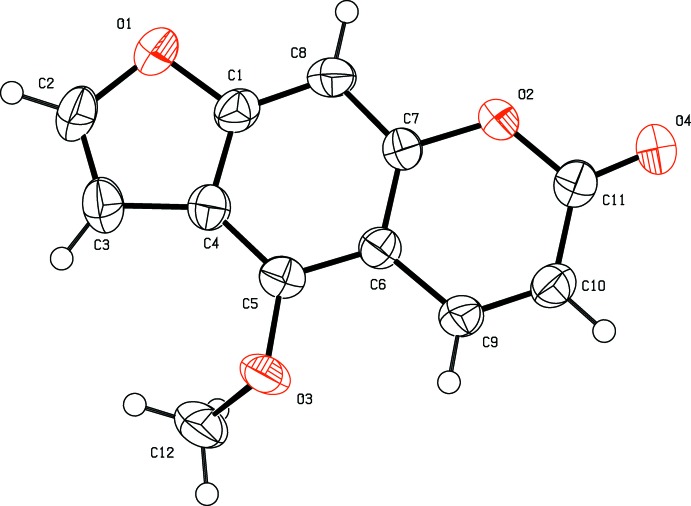
A view of the mol­ecular structure of the title compound, with the atom labelling. Displacement ellipsoids are drawn at the 50% probability level.

The title compound (Fig. 1 ▸), belongs to the psoralen class of compounds and is composed of three fused rings *viz.* furan, benzene and pyrone. It is an almost planar mol­ecule with an r.m.s. deviation of 0.024 Å for the atoms of the fused ring system, O1–O2/C1–C11. The meth­oxy C atom, C12, is displaced from this mean plane by 0.925 (5) Å, while atoms O3 and O4 are displaced from the mean plane by 0.069 (3) and 0.035 (3) Å, respectively.

## Supra­molecular features   

**Figure 2:**
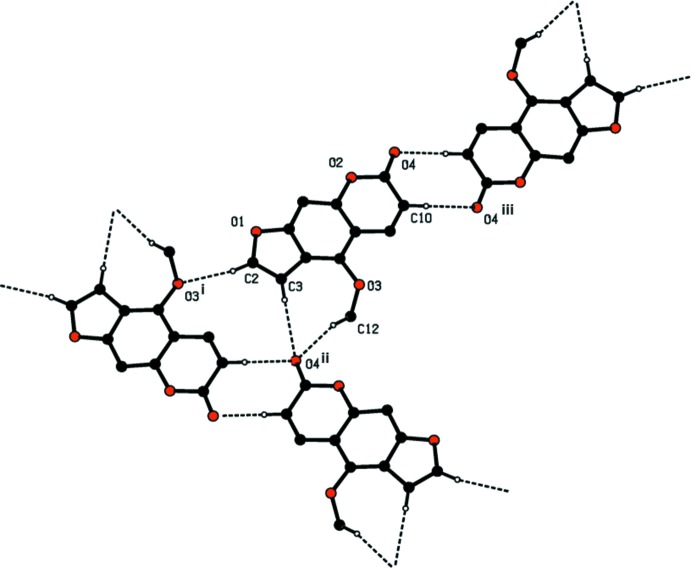
A view of the various C—H⋯O hydrogen bonds (dashed lines; see Table 1 ▸ for details) in the crystal of the title compound.

**Figure 3:**
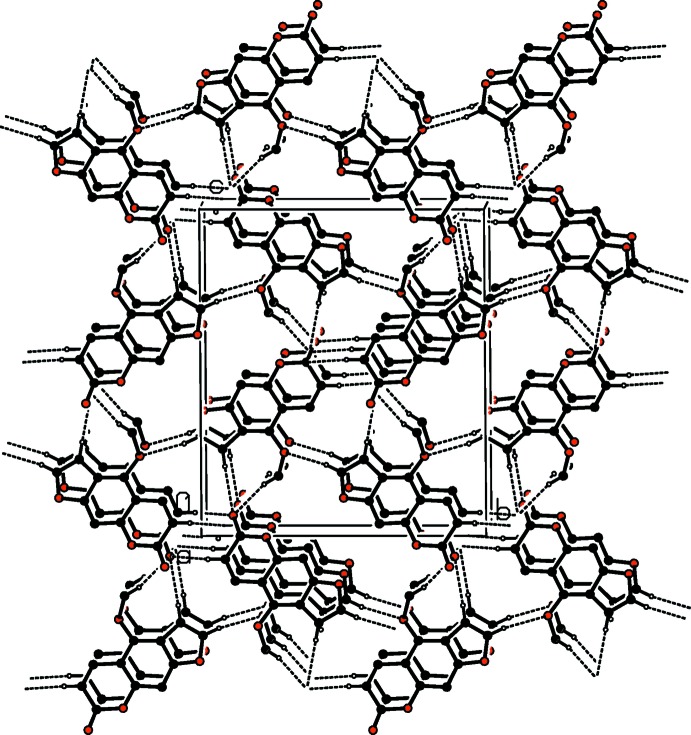
A view along the *a* axis of the crystal packing of the title compound. Hydrogen bonds are drawn as dashed lines (see Table 1 ▸) and H atoms not involved in these inter­actions have been omitted for clarity.

In the crystal, mol­ecules are linked by a series of C—H⋯O hydrogen bonds, which are illustrated in Fig. 2 ▸ (see also Table 1 ▸). They form a three-dimensional network (Table 1 ▸ and Fig. 3 ▸). There are offset π–π inter­actions present involving the coumarin moieties stacking along the *a-*axis direction [shortest inter-centroid distance *Cg*2⋯*Cg*3^i^ = 3.717 (3) Å, inter­planar distance = 3.425 (2) Å, slippage = 1.356 Å, *Cg*2 and *Cg*3 are the centroids of rings O2/C6/C7/C9–C11 and C1/C4–C8, respectively, symmetry code: (i) *x* − 1, *y*, *z*].

## Database survey   

A search of the Cambridge Structural Database (CSD, Version 5.37, last update May 2016; Groom *et al.*, 2016 ▸) gave 16 hits for the furan­ocoumarin skeleton with an O atom substituent in position 5, similar to the title compound. Two compounds closely resemble the title compound, *viz*. 5-hy­droxy­psolalen [JIXBOH; Ginderow, 1991 ▸] isolated from the bark of *Citrus bergamia*, and 5,8-di­meth­oxy­psoralen [ISIMP (293 K); Gopalakrishna *et al.*, 1977 ▸] and [ISIMP01 (120 K); Napolitano *et al.*, 2003 ▸]. The latter was isolated from the roots and leaves of *Adiscanthus fusciflorus (Rutaceae)*.

## Synthesis and crystallization   

The title compound was isolated as a colourless solid from the methanol extract of *T. stictocarpum* by means of column chromatography over silica gel by gradient elution with a mixture of binary solvents system hexane and ethyl acetate. It was purified by reverse phase high-pressure liquid chromatography. Colourless rod-like crystals, suitable crystals for X ray diffraction analysis, were obtained after the title compound was recrystallized three times from ethyl acetate:hexane (1:4) mixed solvents at room temperature by slow evaporation of the solvents (m.p. 469 K).

^1^H NMR data (CHCl_3_, 200 MHz) 8.13 (*d*, 1H, *J* = 9.8 Hz, H-9), 7.57 (*d*, 1H, *J* = 2.2 Hz, H-2), 7.11 (*s*, 1H, H-8), 7.05 (*d*, 1H, *J* = 2.2 Hz, H-3), 6.25 (*d*, 1H, *J* = 9.8 Hz, H-10), 4.26 (*s*, 3H, OCH_3_). EIMS (70 ev) data: *m*/*z* (%) 216 (100; base peak/mol­ecular ion peak) [*M* ^+^], 201 (25.2%) [*M* ^+^−CH_3_), 188 (25.7) [*M* ^+^−OCH_3_], 173 (25.6) [*M* ^+^−(CH_3_–CO)], 145 (33.8) [*M* ^+^−(OCH_3_–CO_2_)], 89(17.0).

## Refinement   

Crystal data, data collection and structure refinement details are summarized in Table 2 ▸. The C-bound H atoms were included in calculated positions and treated as riding atoms: C—H = 0.93–0.96 Å with *U*_iso_(H) = 1.5*U*_eq_(C-meth­yl) and 1.2*U*eq(C) for other H atoms. The structure was refined as a two-component twin [180° rotation about the a* axis; BASF = 0.3955 (2)].

## Supplementary Material

Crystal structure: contains datablock(s) I, Global. DOI: 10.1107/S2056989016011221/su5310sup1.cif

Structure factors: contains datablock(s) I. DOI: 10.1107/S2056989016011221/su5310Isup2.hkl

Supporting information file. DOI: 10.1107/S2056989016011221/su5310Isup3.cml

CCDC reference: 1491854

Additional supporting information: crystallographic information; 3D view; checkCIF report

## Figures and Tables

**Table 1:** Hydrogen-bond geometry (Å, °)

*D*—H⋯*A*	*D*—H	H⋯*A*	*D*⋯*A*	*D*—H⋯*A*
C2—H2⋯O3^i^	0.93	2.49	3.406 (5)	170
C3—H3⋯O4^ii^	0.93	2.57	3.484 (6)	170
C10—H10⋯O4^iii^	0.93	2.51	3.387 (5)	158
C12—H12*A*⋯O4^ii^	0.96	2.44	3.376 (5)	165

Symmetry codes: (i) 
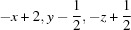
; (ii) 
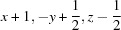
; (iii) 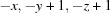.

**Table 2:** Experimental details

Crystal data	
Chemical formula	C_12_H_8_O_4_
*M*_r_	216.18
Crystal system, space group	Monoclinic, *P*2_1_/*c*
Temperature (K)	299
*a*, *b*, *c* (Å)	3.8486 (8), 14.676 (2), 16.866 (3)
β (°)	92.12 (2)
*V* (Å^3^)	952.0 (3)
*Z*	4
Radiation type	Mo *K*α
μ (mm^−1^)	0.12
Crystal size (mm)	0.44 × 0.08 × 0.02

Data collection	
Diffractometer	Oxford Diffraction Xcalibur with a Sapphire CCD detector
Absorption correction	Multi-scan (*CrysAlis RED*; Oxford Diffraction, 2009 ▸)’
*T*_min_, *T*_max_	0.951, 0.998
No. of measured, independent and observed [*I* > 2σ(*I*)] reflections	7096, 7096, 3811
*R*_int_	0.08
(sin θ/λ)_max_ (Å^−1^)	0.602

Refinement	
*R*[*F* ^2^ > 2σ(*F* ^2^)], *wR*(*F* ^2^), *S*	0.055, 0.138, 0.86
No. of reflections	7096
No. of parameters	147
H-atom treatment	H-atom parameters constrained
Δρ_max_, Δρ_min_ (e Å^−3^)	0.19, −0.22

Computer programs: *CrysAlis CCD* and *CrysAlis RED* (Oxford Diffraction, 2009 ▸), *SHELXS2014* (Sheldrick, 2008 ▸), *SHELXL2014* (Sheldrick, 2015 ▸) and *PLATON* (Spek, 2009 ▸).

## supplementary crystallographic information

### Crystal data

**Table:**

C_12_H_8_O_4_	*D*_x_ = 1.508 Mg m^−^^3^
*M**_r_* = 216.18	Melting point: 469 K
Monoclinic, *P*2_1_/*c*	Mo *K*α radiation, λ = 0.71073 Å
*a* = 3.8486 (8) Å	Cell parameters from 870 reflections
*b* = 14.676 (2) Å	θ = 2.8–27.9°
*c* = 16.866 (3) Å	µ = 0.12 mm^−^^1^
β = 92.12 (2)°	*T* = 299 K
*V* = 952.0 (3) Å^3^	Needle, colourless
*Z* = 4	0.44 × 0.08 × 0.02 mm
*F*(000) = 448	

### Data collection

**Table:**

Oxford Diffraction Xcalibur with a Sapphire CCD detector diffractometer	7096 independent reflections
Radiation source: fine-focus sealed tube	3811 reflections with *I* > 2σ(*I*)
Graphite monochromator	*R*_int_ = 0.08
Rotation method data acquisition using ω and phi scans.	θ_max_ = 25.4°, θ_min_ = 2.8°
Absorption correction: multi-scan (CrysAlis RED; Oxford Diffraction, 2009)'	*h* = −4→4
*T*_min_ = 0.951, *T*_max_ = 0.998	*k* = −17→17
7096 measured reflections	*l* = −20→20

### Refinement

**Table:**

Refinement on *F*^2^	Primary atom site location: structure-invariant direct methods
Least-squares matrix: full	Secondary atom site location: difference Fourier map
*R*[*F*^2^ > 2σ(*F*^2^)] = 0.055	Hydrogen site location: inferred from neighbouring sites
*wR*(*F*^2^) = 0.138	H-atom parameters constrained
*S* = 0.86	*w* = 1/[σ^2^(*F*_o_^2^) + (0.0738*P*)^2^] where *P* = (*F*_o_^2^ + 2*F*_c_^2^)/3
7096 reflections	(Δ/σ)_max_ = 0.002
147 parameters	Δρ_max_ = 0.19 e Å^−^^3^
0 restraints	Δρ_min_ = −0.22 e Å^−^^3^

### Special details

**Table:**

Geometry. All esds (except the esd in the dihedral angle between two l.s. planes) are estimated using the full covariance matrix. The cell esds are taken into account individually in the estimation of esds in distances, angles and torsion angles; correlations between esds in cell parameters are only used when they are defined by crystal symmetry. An approximate (isotropic) treatment of cell esds is used for estimating esds involving l.s. planes.
Refinement. Refined as a 2-component twin.

### Fractional atomic coordinates and isotropic or equivalent isotropic displacement parameters (Å^2^)

**Table:**

	*x*	*y*	*z*	*U*_iso_*/*U*_eq_	
O1	0.7447 (9)	0.00489 (18)	0.37565 (18)	0.0525 (10)	
O2	0.3156 (8)	0.26727 (18)	0.50579 (14)	0.0399 (9)	
O3	0.7860 (9)	0.28906 (17)	0.24893 (16)	0.0510 (10)	
O4	0.1046 (9)	0.3863 (2)	0.56614 (18)	0.0617 (11)	
C1	0.6682 (13)	0.0956 (3)	0.3822 (3)	0.0392 (13)	
C2	0.8854 (13)	−0.0043 (3)	0.3016 (3)	0.0534 (15)	
H2	0.9619	−0.0595	0.2814	0.064*	
C3	0.8989 (13)	0.0740 (3)	0.2626 (3)	0.0477 (14)	
H3	0.9832	0.0833	0.2123	0.057*	
C4	0.7573 (13)	0.1417 (3)	0.3137 (2)	0.0369 (12)	
C5	0.6975 (12)	0.2354 (3)	0.3111 (2)	0.0344 (12)	
C6	0.5523 (11)	0.2781 (3)	0.3757 (2)	0.0304 (11)	
C7	0.4677 (11)	0.2262 (3)	0.4418 (2)	0.0348 (12)	
C8	0.5234 (12)	0.1339 (3)	0.4477 (3)	0.0397 (13)	
H8	0.4679	0.1003	0.4922	0.048*	
C9	0.4692 (12)	0.3738 (3)	0.3771 (3)	0.0373 (13)	
H9	0.5221	0.4101	0.3339	0.045*	
C10	0.3191 (12)	0.4114 (3)	0.4385 (2)	0.0431 (13)	
H10	0.2654	0.4732	0.4371	0.052*	
C11	0.2370 (13)	0.3592 (3)	0.5074 (3)	0.0433 (13)	
C12	0.6549 (14)	0.2653 (3)	0.1726 (2)	0.0652 (17)	
H12A	0.7936	0.2173	0.1516	0.098*	
H12B	0.6630	0.3175	0.1384	0.098*	
H12C	0.4187	0.2450	0.1758	0.098*	

### Atomic displacement parameters (Å^2^)

**Table:**

	*U*^11^	*U*^22^	*U*^33^	*U*^12^	*U*^13^	*U*^23^
O1	0.075 (3)	0.0265 (17)	0.056 (2)	0.0074 (19)	0.006 (2)	−0.0024 (16)
O2	0.054 (2)	0.0343 (18)	0.0321 (16)	0.0034 (18)	0.0073 (19)	0.0003 (14)
O3	0.081 (3)	0.0419 (18)	0.0300 (17)	−0.0206 (19)	0.005 (2)	0.0006 (15)
O4	0.089 (3)	0.052 (2)	0.045 (2)	0.018 (2)	0.025 (2)	−0.0027 (18)
C1	0.045 (4)	0.029 (3)	0.043 (3)	0.000 (3)	−0.007 (3)	0.000 (2)
C2	0.063 (4)	0.040 (3)	0.058 (3)	0.009 (3)	0.010 (3)	−0.013 (3)
C3	0.054 (4)	0.041 (3)	0.048 (3)	−0.001 (3)	0.007 (3)	−0.006 (2)
C4	0.040 (3)	0.032 (3)	0.039 (3)	−0.002 (3)	−0.001 (3)	−0.0073 (19)
C5	0.036 (3)	0.037 (3)	0.030 (2)	−0.006 (3)	0.001 (3)	0.001 (2)
C6	0.029 (3)	0.029 (2)	0.033 (2)	−0.002 (2)	−0.002 (2)	−0.001 (2)
C7	0.041 (3)	0.033 (3)	0.031 (2)	−0.002 (3)	0.005 (2)	−0.002 (2)
C8	0.049 (4)	0.033 (3)	0.038 (3)	−0.002 (3)	0.004 (3)	0.008 (2)
C9	0.047 (3)	0.030 (3)	0.035 (3)	−0.004 (2)	0.002 (3)	0.005 (2)
C10	0.056 (4)	0.029 (2)	0.044 (3)	0.004 (3)	0.001 (3)	0.003 (2)
C11	0.046 (4)	0.036 (3)	0.048 (3)	0.007 (3)	0.006 (3)	−0.001 (2)
C12	0.099 (5)	0.060 (3)	0.037 (3)	−0.013 (4)	0.001 (3)	−0.003 (2)

### Geometric parameters (Å, º)

**Table:**

O1—C1	1.369 (5)	C4—C5	1.394 (6)
O1—C2	1.386 (5)	C5—C6	1.391 (5)
O2—C7	1.385 (4)	C6—C7	1.398 (5)
O2—C11	1.383 (5)	C6—C9	1.442 (5)
O3—C5	1.365 (4)	C7—C8	1.374 (5)
O3—C12	1.409 (4)	C8—H8	0.9300
O4—C11	1.200 (5)	C9—C10	1.325 (5)
C1—C8	1.376 (5)	C9—H9	0.9300
C1—C4	1.393 (6)	C10—C11	1.435 (5)
C2—C3	1.327 (6)	C10—H10	0.9300
C2—H2	0.9300	C12—H12A	0.9600
C3—C4	1.435 (6)	C12—H12B	0.9600
C3—H3	0.9300	C12—H12C	0.9600
			
C1—O1—C2	105.1 (3)	C8—C7—O2	116.2 (4)
C7—O2—C11	122.6 (3)	C8—C7—C6	123.7 (4)
C5—O3—C12	117.9 (3)	O2—C7—C6	120.1 (4)
O1—C1—C8	123.8 (4)	C1—C8—C7	114.2 (4)
O1—C1—C4	110.2 (4)	C1—C8—H8	122.9
C8—C1—C4	126.0 (4)	C7—C8—H8	122.9
C3—C2—O1	112.7 (4)	C10—C9—C6	121.4 (4)
C3—C2—H2	123.7	C10—C9—H9	119.3
O1—C2—H2	123.7	C6—C9—H9	119.3
C2—C3—C4	106.2 (4)	C9—C10—C11	121.7 (4)
C2—C3—H3	126.9	C9—C10—H10	119.1
C4—C3—H3	126.9	C11—C10—H10	119.1
C5—C4—C1	117.3 (4)	O4—C11—O2	116.1 (4)
C5—C4—C3	136.9 (4)	O4—C11—C10	127.2 (4)
C1—C4—C3	105.8 (4)	O2—C11—C10	116.8 (4)
O3—C5—C6	117.4 (4)	O3—C12—H12A	109.5
O3—C5—C4	123.2 (4)	O3—C12—H12B	109.5
C6—C5—C4	119.4 (4)	H12A—C12—H12B	109.5
C5—C6—C7	119.4 (4)	O3—C12—H12C	109.5
C5—C6—C9	123.2 (4)	H12A—C12—H12C	109.5
C7—C6—C9	117.4 (4)	H12B—C12—H12C	109.5
			
C2—O1—C1—C8	−179.8 (5)	C4—C5—C6—C9	−177.6 (4)
C2—O1—C1—C4	0.3 (5)	C11—O2—C7—C8	−178.5 (4)
C1—O1—C2—C3	−0.2 (6)	C11—O2—C7—C6	0.9 (6)
O1—C2—C3—C4	0.0 (6)	C5—C6—C7—C8	0.8 (6)
O1—C1—C4—C5	−179.6 (4)	C9—C6—C7—C8	178.3 (4)
C8—C1—C4—C5	0.5 (7)	C5—C6—C7—O2	−178.5 (4)
O1—C1—C4—C3	−0.3 (5)	C9—C6—C7—O2	−1.1 (6)
C8—C1—C4—C3	179.8 (5)	O1—C1—C8—C7	−179.9 (5)
C2—C3—C4—C5	179.2 (6)	C4—C1—C8—C7	−0.1 (7)
C2—C3—C4—C1	0.2 (6)	O2—C7—C8—C1	178.7 (4)
C12—O3—C5—C6	−126.8 (4)	C6—C7—C8—C1	−0.6 (7)
C12—O3—C5—C4	55.5 (6)	C5—C6—C9—C10	177.4 (5)
C1—C4—C5—O3	177.3 (4)	C7—C6—C9—C10	0.1 (7)
C3—C4—C5—O3	−1.7 (9)	C6—C9—C10—C11	1.1 (7)
C1—C4—C5—C6	−0.3 (7)	C7—O2—C11—O4	179.7 (4)
C3—C4—C5—C6	−179.3 (5)	C7—O2—C11—C10	0.3 (6)
O3—C5—C6—C7	−178.0 (4)	C9—C10—C11—O4	179.3 (5)
C4—C5—C6—C7	−0.3 (6)	C9—C10—C11—O2	−1.3 (7)
O3—C5—C6—C9	4.7 (6)		

### Hydrogen-bond geometry (Å, º)

**Table:**

*D*—H···*A*	*D*—H	H···*A*	*D*···*A*	*D*—H···*A*
C2—H2···O3^i^	0.93	2.49	3.406 (5)	170
C3—H3···O4^ii^	0.93	2.57	3.484 (6)	170
C10—H10···O4^iii^	0.93	2.51	3.387 (5)	158
C12—H12*A*···O4^ii^	0.96	2.44	3.376 (5)	165

Symmetry codes: (i) −*x*+2, *y*−1/2, −*z*+1/2; (ii) *x*+1, −*y*+1/2, *z*−1/2; (iii) −*x*, −*y*+1, −*z*+1.
